# Prevalence, attitudes, behaviours and policy evaluation of midwakh smoking among young people in the United Arab Emirates: Cross-sectional analysis of the Global Youth Tobacco Survey

**DOI:** 10.1371/journal.pone.0215899

**Published:** 2019-04-24

**Authors:** Mohammed Jawad, Mohammed Al-Houqani, Raghib Ali, Yehya El Sayed, Omar ElShahawy, Michael Weitzman, Scott E. Sherman

**Affiliations:** 1 Public Health Policy Evaluation Unit, Imperial College London, London, United Kingdom; 2 College of Medicine and Health Sciences, UAE University, Abu Dhabi, United Arab Emirates; 3 Public Health Research Center, New York University Abu Dhabi, Abu Dhabi, United Arab Emirates; 4 College of Arts and Sciences, American University of Sharjah, Sharjah, United Arab Emirates; 5 Department of Population Health, New York University School of Medicine, New York, New York, United States of America; 6 College of Global Public Health, New York University, New York, New York, United States of America; 7 AHA Tobacco Regulation and Addiction Center, American Heart Association, Dallas, Texas, United States of America; 8 Departments of Pediatrics and Environmental Medicine, New York University School of Medicine, New York, New York, United States of America; University of Calfornia San Francisco, UNITED STATES

## Abstract

**Introduction:**

Non-cigarette tobacco products are an increasing public health concern globally. Little is known about midwakh, a pipe indigenous to the United Arab Emirates (UAE). This study aimed to assess the prevalence, attitudes, behaviours and policy evaluation of midwakh smoking among 13 to 15 year olds in the UAE.

**Methods:**

We conducted secondary analyses of the 2013 UAE Global Youth Tobacco Survey. The main three outcomes were ever use, current use (past-30 days), and the number of midwakhs smoked per day. We assessed cessation, attitude, and policy measures. Regression models identified the association between each outcome measure and sex, school grade, nationality, weekly spending money, cigarette use, and parent and peer tobacco use.

**Results:**

The prevalence of ever and current midwakh use were 18.5% and 9.0%, respectively. Daily midwakh users smoked a median of 8.0 per day while non-daily users smoked 3.8 per month. Higher midwakh prevalence was reported among wealthier males, older age groups, concurrent cigarette users and among participants having peers or parents who use tobacco. There was also variation by nationality. Reduced harm perception was greater among midwakh users than non-users. About 39.6% reported being declined a midwakh purchase due to age, and 35.5% reported noticing health warnings on packages.

**Conclusions:**

Midwakh use is prevalent among 13 to 15 year olds in the UAE, and burden lies mainly with daily users. Further needed research should not delay implementation and evaluation of policies known to curb tobacco use among youth, including taxation, media campaigns, and provision of cessation services.

## Introduction

The use of non-cigarette tobacco products has grown substantially in recent decades [1]. About 36% of global tobacco consumers use non-cigarette tobacco products exclusively [2]. In India, bidis are as prevalent as manufactured cigarettes [3] and in the US, the value of non-cigarette tobacco product shipments almost doubled between 2000 and 2013, from $4 billion to $7.7 billion [4]. The Middle East has seen a resurgence in waterpipe tobacco use [5], where in many settings it has exceeded the prevalence of cigarette use [6]. Waterpipe use has also increased in many countries outside the Middle East [5], creating a global public health worry.

A less reported non-cigarette tobacco product is midwakh, which has recently emerged as a public health concern in the United Arab Emirates (UAE). Midwakh is a narrow pipe used to smoke dried tobacco leaves mixed with herbs, spices, or barks known as “dokha” (Arabic for “dizzy”) [7]. Three main categories of dokha blends are commonly used: cold (light), warm (medium) and hot (strong) dokha. They likely refer to the harshness/strength levels of the buzz rather than the amount of dohka smoke [7, 8]. A few deep inhalations are required to consume the dokha placed in the bowl of the pipe [9]. In the UAE, a week’s supply of midwakh is about seven times cheaper than cigarettes (3 USD vs. 21 USD).[9]

While there is reason to be concerned that midwakh use is harmful to health, a full toxicological assessment is lacking and there is a paucity of epidemiological data. Anecdotal reports suggest a high level of nicotine is responsible for the “dizziness” description ascribed to this tobacco product. This hypothesis supports the findings of one pre-post study among 97 dokha users which documented an increase in systolic blood pressure, heart rate, and respiratory rate after midwakh use [10]. It also is possible that the high nicotine content of midwakh was responsible for seizures in seven otherwise healthy 14 to 27 year olds with no history of other drug use, and in all of whom the seizures resolved after midwakh cessation [11]. Further, a recent chemical analysis of dokha tobacco found levels of nitrogen, heavy metals and other compounds known to be harmful to human health including carcinogens, central nervous system depressants, and irritants [12].

All prevalence studies of midwakh use to date are from the UAE and show a predominance of use among males, although the variation in epidemiological measures used and populations surveyed makes comparisons difficult. One national screening programme conducted between 2009 and 2010 reported that 1.7% of adults are daily midwakh user, [9] while another conducted in 2011 reported 12.0% of men used midwakh some days or everyday [13]. The baseline characteristics of one cohort study that used convenience sampling in 2015 reported that 13.0% of adults used midwakh currently [14]. The prevalence of midwakh, however, appears higher among students. For example, one small study among university students in Ajman reported 11.5% ever midwakh use [15]. School-based studies reported ever use between 12.9% and 36.3%, and current use between 8.5% and 24.1% [16, 17], but none of these studies were representative of the UAE population and both had small sample sizes.

In light of the lack of nationally representative prevalence data among young people in UAE, there is a need to better understand the epidemiology of midwakh use among adolescents. Furthermore, there are clear research gaps in the behaviours and attitudes related to midwakh, and the role of existing policy measures in preventing its use and promoting cessation. This study, therefore, aimed to assess the prevalence, attitudes, behaviours and policy implications of midwakh smoking among 13 to 15 year olds in the UAE.

## Methods

### Design

We conducted secondary analyses of the 2013 UAE Global Youth Tobacco Survey (GYTS); a self-administered, anonymous school-based survey that uses a two stage cluster design to produce nationally-representative estimates of tobacco use. Schools were chosen with probability proportional to their size and the classes were selected with equal probability. The target age range for GYTS is 13 to 15 years. UAE added an optional midwakh module to the existing GYTS questionnaire. More methodological details on the GYTS can be found elsewhere [18].

### Measures

Outcome measures included prevalence measures (ever use, current [past-30 day] use, daily use, number of times midwakh was smoked per day or month, age of first use), cessation measures (intention to quit, quit attempts), attitudes (reduced perception of susceptibility to second-hand smoke, increased peer-influenced propensity to smoke, increased perceived ease of quitting, increased perceived comfort, increased perceived enjoyment) accessibility (attainment method, smoking location), and policy measures (refusal of sale due to age, health warning recall). The exact wording of these questions, and our corresponding definitions, are outlined in S1 Table. Importantly, questions regarding cessation measures included the option “I don’t smoke now”, so answers are limited to a sub-group who consider themselves “active users”; a different construct to past-30 day use. Independent variables included sex, school grade (8, 9, or 10), nationality, weekly spending money, current (past-30 day) and current cigarette use, and parent and peer tobacco use status.

### Analysis

To ensure consistency across responses and to minimise the number of missing values, we created a validation rule whereby any respondent who answered negatively to ever smoking midwakh would automatically have their remaining answers recoded to that of a never midwakh user. The degree of missing data varied from 0.3% to 4.1% for each variable of interest; a listwise deletion would have resulted in 14.5% of observations being removed. Observations with missing data did not significantly differ to those without missing data with respect to sex, grade, nationality, and weekly spending money (p<0.05).

We analysed our sample descriptively using weighted percentages, and conducted bivariate analysis between prevalence outcome measures (ever use, current use, number of midwakh smoked per day or per month) and independent variables. We classified behavioural attitudes by midwakh status (current, ever, never) to identify sub-group patterns. We constructed regression models to test the adjusted association between independent variables and ever and current use (logistic regression) and number of midwakh smoked per month (linear regression–among dokha users only). We logarithmically transformed the variable “midwakhs per month” prior to regression to account for its skewness. For the bivariate and multivariate analyses only, we performed multiple imputation to account for any selection bias resulting from missing data. The data were considered to have an arbitrary missing data pattern and we performed multivariate imputation using chained equations. The chained equation approach imputes multiple variables iteratively through a sequence of univariate imputation models, one for each imputation variable, with fully conditional specifications of prediction equations [19]. Five imputation cycles were sufficient to reach convergence. An alpha value of 5% was used to determine statistical significance. Sample weights were utilised to account for the complex design of the GYTS and we reported adjusted odds ratios (AOR) with 95% confidence intervals (95% CI). All analyses were conducted on Stata 15.0 (StataCorp).

## Results

### Sample characteristics

The characteristics of our sample (N = 4,259) are shown in Table 1. In general, the sample was evenly weighted by sex and by grade, 40.1% were UAE nationals, and 64.9% had less than 19 USD of weekly spending money. Approximately a quarter (26.6%) had ever tried cigarettes, and 7.9% were past-30 day users. Just over a fifth (23.3%) had at least one parent who smoked tobacco, and 36.4% had some, most, or all friends who smoked tobacco.

**Table 1:** Sample characteristics (N = 4,259).

Characteristic	N	Weighted %
**Sex**		
Male	*1*,*916*	*52*.*9*
Female	*2*,*322*	*47*.*1*
**School grade**		
8	*1*,*556*	*35*.*4*
9	*1*,*333*	*33*.*0*
10	*1*,*333*	*31*.*7*
**Nationality**		
UAE	*1*,*990*	*40*.*1*
South East Asia	*871*	*24*.*4*
Levant (Lebanon, Syria, Jordan, Palestine, Iraq)	*503*	*13*.*3*
North Africa (Egypt, Sudan, Tunisia, Libya, Morocco, Algeria, Mauritania)	*353*	*9*.*1*
Gulf (Kuwait, Saudi Arabia, Oman, Qatar, Bahrain, Yemen)	*255*	*5*.*6*
Europe, USA, Australia	*112*	*3*.*2*
Other	*157*	*4*.*3*
Weekly spending money*		
I usually don’t have any spending money	*633*	*16*.*0*
Less than 8 USD	*1*,*009*	*23*.*7*
8–19 USD	*1*,*082*	*25*.*2*
19–35 USD	*771*	*18*.*3*
35 USD and more	*750*	*16*.*9*
**Current cigarette use**		
No	*3*,*834*	*92*.*1*
Yes	*307*	*7*.*9*
**Parent tobacco status**		
Neither smoke	*3*,*229*	*76*.*7*
At least one parent smokes	*992*	*23*.*3*
**Peer tobacco status**		
None smoke	*2*,*761*	*63*.*5*
At least some friends smoke	*1*,*401*	*36*.*4*

*Conversion rate in 2013: 1 dh = 0.2722 USD, estimates rounded to nearest US dollar; variables do not total to 4,259 due to missing data

### Midwakh use

Prevalence of ever and current midwakh use were 18.5% (95% CI 17.3–19.7) and 9.0% (95% CI 8.1–9.9), respectively. Of current users, 15.9% smoked midwakh daily (19.6% among males and 4.8% among females) and the remainder smoked on a non-daily basis. Daily midwakh users smoked a median of 8.0 midwakhs per day (interquartile range (IQR) 3.5–15.5), and non-daily midwakh users smoked 3.8 midwakhs per month (IQR 1.5–14.0). Among ever midwakh users, the median age of first midwakh use was 12.5 years (IQR 8.5–14.5); this was similar between non-current midwakh users (12.5 [IQR 8.5–14.5]) and current midwakh users (12.5 [IQR 10.5–14.5], p = 0.089). Among those who reported ever having used midwakh, 65.3% had also tried cigarettes, and of all current midwakh users 56.3% were current cigarette users (i.e. dual current users of both midwakh and cigarettes).

Table 2 presents results of the bivariate and multivariate analysis following multiple imputation. The prevalence of ever having used midwakh was almost twice as high in males as in females; a relationship that remained when adjusted for other factors in the multivariate analysis. Ever midwakh use increased in a stepwise manner by grade and weekly spending money, and those of UAE nationality had significantly higher use than all other nationalities except for those from Gulf, Europe/USA/Australia, and “other” countries. Furthermore, ever midwakh use was significantly higher among current cigarette users, and among those who reported peer and parent tobacco use.

**Table 2:** Bivariate and multivariate analysis showing the association between sociodemographic variables and midwakh use.

Characteristic	Ever midwakh use (n = 790)		Current midwakh use (n = 376)		Midwakhs per month (n = 451)	
	Weighted %	AOR (95% CI)	Weighted %	AOR (95% CI)	Median	B coefficient (95% CI)
**Sex**						
Male	*21*.*0*	*1*.*00*	*9*.*7*	*1*.*00*	*5*.*3*	*0*.*00*
Female	*12*.*5*	*0*.*77 (0*.*63*, *0*.95)*	*3*.*4*	*0*.*64 (0*.*46*, *0*.89)**	*4*.*0*	*-0*.*42 (-0*.*90*, *0*.*06)*
**School grade**						
8	*12*.*5*	*1*.*00*	*4*.*8*	*1*.*00*	*5*.*3*	*0*.*00*
9	*17*.*2*	*1*.*43 (1*.*13*, *1*.81)**	*6*.*9*	*1*.*45 (0*.*98*, *2*.*14)*	*4*.*0*	*-0*.*44 (-1*.*00*, *0*.*11)*
10	*21*.*6*	*1*.*56 (1*.*24*, *1*.97)***	*8*.*6*	*1*.*30 (0*.*89*, *1*.*91)*	*5*.*3*	*-0*.*05 (-0*.*59*, *0*.*49)*
**Nationality**						
UAE	*20*.*9*	*1*.*00*	*8*.*6*	*1*.*00*	*4*.*0*	*0*.*00*
South East Asia	*8*.*7*	*0*.*48 (0*.*36*, *0*.65)***	*1*.*9*	*0*.*38 (0*.*24*, *0*.62)***	*5*.*3*	*0*.*50 (-0*.*33*, *1*.*32)*
Levant	*19*.*6*	*0*.*71 (0*.*53*, *0*.95)*	*6*.*9*	*0*.*46 (0*.*29*, *0*.75)**	*7*.*3*	*-0*.*12 (-0*.*80*, *0*.*57)*
North Africa	*13*.*3*	*0*.*58 (0*.*40*, *0*.84)**	*7*.*6*	*0*.*97 (0*.*60*, *1*.*58)*	*4*.*0*	*0*.*24 (-0*.*61*, *1*.*09)*
Gulf	*21*.*6*	*1*.*02 (0*.*69*, *1*.*50)*	*11*.*5*	*1*.*31 (0*.*78*, *2*.*19)*	*4*.*0*	*0*.*38 (-0*.*29*, *1*.*05)*
Europe, USA, Australia	*19*.*4*	*0*.*60 (0*.*35*, *1*.*02)*	*10*.*1*	*0*.*55 (0*.*24*, *1*.*23)*	*14*.*0*	*0*.*30 (-0*.*93*, *1*.*54)*
Other	*17*.*8*	*0*.*83 (0*.*51*, *1*.*35)*	*6*.*0*	*0*.*72 (0*.*35*, *1*.*50)*	*23*.*3*	*0*.*04 (-0*.*81*, *0*.*90)*
**Weekly spending money**						
I usually don’t have any spending money	*8*.*5*	*1*.*00*	*3*.*0*	*1*.*00*	*4*.*0*	*0*.*00*
Less than 8 USD	*11*.*2*	*1*.*09 (0*.*75*, *1*.*58)*	*3*.*7*	*0*.*86 (0*.*48*, *1*.*54)*	*2*.*0*	*-0*.*38 (-1*.*43*, *0*.*67)*
8–19 USD	*19*.*5*	*1*.*76 (1*.*24*, *2*.50)**	*7*.*1*	*1*.*33 (0*.*76*, *2*.*32)*	*5*.*3*	*-0*.*39 (-1*.*37*, *0*.*60)*
19–35 USD	*19*.*4*	*1*.*52 (1*.*06*, *2*.17)*	*8*.*3*	*1*.*36 (0*.*76*, *2*.*43)*	*5*.*3*	*-0*.*07 (-1*.*03*, *0*.*90)*
35 USD and more	*26*.*9*	*2*.*33 (1*.*60*, *3*.37)***	*12*.*5*	*2*.*13 (1*.*21*, *3*.76)**	*14*.*0*	*0*.*04 (-0*.*81*, *0*.*90)*
**Current cigarette use**						
No	*13*.*6*	*1*.*00*	*3*.*5*	*1*.*00*	*3*.*8*	*0*.*00*
Yes	*65*.*2*	*9*.*63 (6*.*94*, *13*.34)***	*52*.*2*	*22*.*58 (16*.*1*, *31*.7)***	*12*.*3*	*0*.*79 (0*.*30*, *1*.28)**
**Parent tobacco status**						
Neither smoke	*14*.*0*	*1*.*00*	*4*.*2*	*1*.*00*	*5*.*3*	*0*.*00*
At least one parent smokes	*26*.*8*	*1*.*48 (1*.*15*, *1*.89)**	*15*.*0*	*2*.*21 (1*.*61*, *3*.02)***	*4*.*0*	*-0*.*06 (-0*.*51*, *0*.*38)*
**Peer tobacco status**						
None smoke	*10*.*1*	*1*.*00*	*2*.*4*	*1*.*00*	*2*.*0*	*0*.*00*
At least some friends smoke	*29*.*3*	*2*.*49 (2*.*01*, *3*.*07)*	*14*.*3*	*3*.*40 (2*.*42*, *4*.77)***	*7*.*5*	*1*.*00 (0*.*48*, *1*.51)***

*p<0.05

**p<0.01

***p<0.001; regression results are adjusted for all variables found in the table; ever and current midwakh use analysed among the full sample, midwakhs per month analysed among dokha users only

Significant correlates for current midwakh use were qualitatively similar to ever midwakh use with respect to sex, nationality current cigarette use, and among those who reported peer and parent tobacco use. While correlates of current midwakh use with respect to grade and weekly spending money were in a similar effect direction to those for ever midwakh use, these did not reach statistical significance in the multivariate analysis.

### Behavioural and policy attitudes

Among respondents who considered themselves midwakh users at the time of the survey, 48.2% wanted to stop smoking midwakh now and 55.4% made at least a quit attempt within the past year. Among never users of midwakh, 36.6% reported increased perceived enjoyment should they try midwakh.

Table 3 provides the breakdown of behavioural attitudes, which were asked among the full sample, by midwakh use status. In general, current and ever midwakh users had a greater reduced harm perception than non-users, with current users reporting the most reduced harm perception. For example, 23.1% of all respondents thought that the smoke from other people’s midwakh was definitely or probably not harmful; this figure was 44.8% among current midwakh users and 34.9% among ever midwakh users. Other attitudes are reported in Table 3.

**Table 3:** Behavioural attitudes stratified by midwakh use status, %.

Behavioural attitude	Full sample (N = 3,643)	Current midwakh use (n = 228)	Ever midwakh use (n = 587)	Never midwakh use (n = 3,056)
**Thinks the smoke from other people’s midwakh is harmful**				
Probably or definitely yes	*76*.*9*	*55*.*2*	*65*.*1*	*80*.*7*
Probably or definitely not	*23*.*1*	*44*.*8*	*34*.*9*	*19*.*3*
**Would smoke midwakh if one of their best friends offered**				
Probably or definitely not	*86*.*8*	*36*.*4*	*58*.*9*	*93*.*6*
Probably or definitely yes	*13*.*2*	*63*.*6*	*41*.*1*	*6*.*4*
**Thinks it would be difficult for someone to quit smoking midwakh once they have started**				
Probably or definitely yes	*67*.*4*	*56*.*8*	*58*.*1*	*70*.*3*
Probably or definitely not	*32*.*6*	*43*.*2*	*41*.*9*	*29*.*7*

Note: current midwakh users are included in the definition of ever midwakh use

Among current (past-30 day) midwakh users, 34.1% reported obtaining dokha tobacco from someone else, 26.9% from a “midwakh shop” or grocery store, and 15.9% from some other way. Furthermore, among current midwakh users, the smoking location was most often reported as “other” (44.5%), followed by at home (25.3%), at a coffee shop (14.3%), and at school (7.7%).

Among those who tried to buy midwakh in the preceding 30 days, 39.6% reported being refused a midwakh purchase due to their age. Among the full sample, 35.5% reported seeing health warnings on dokha tobacco packages, of which 76.3% (27.1% of the full sample) stated it led them to think about quitting or not starting midwakh.

## Discussion

### Summary of findings

In this nationally-representative, cross-sectional study of over 4,200 students from the UAE, about one in five had tried midwakh and one in 11 were current users. In general, midwakh use was higher among wealthier males, older age groups, concurrent cigarette users and among those whose peers or parents used tobacco. There was variation by nationality as well; UAE, Gulf nationals, and those from Europe/USA/Australia had higher use than other nationalities. The burden of midwakh use lies firmly with daily users, who used it about 60 times more often per month than non-daily users. Midwakh users had greater reduced harm perception towards midwakh than non-users. There is large potential for policy improvements, including prohibiting underage sales and ensuring health warning recall.

### Comparison to the literature

Our documented ever use prevalence (18.7%) falls in line with the literature for this age group (12.9–36.3%) [16, 17]. Our daily midwakh users smoked slightly less frequently than adults who smoked daily (8.0 vs. 12.1 per day) [9], and more frequently than a smaller study of students in Dubai (6.4 per day) [20], although the wide measures of precision indicate the patterns of use vary quite substantially between, and possibly within, individual users. Other prevalence studies report similar findings with respect to midwakh use being higher among males [9, 14, 15, 17] and older teenage age groups [16], although in one large study among adults the average age of midwakh users was several years lower than other tobacco users [9]. To our knowledge only one prior study has explored attitudes towards midwakh, a small study of 96 adults of whom half smoked midwakh daily, and belief in being able to quit midwakh was much higher than our reported estimate among current users (73.2% vs. 42.3%) [10].

### Strengths and weaknesses

This study’s merit lies in its large sample size and depth of midwakh questions that allow for a more insightful understanding of midwakh epidemiology. It is the first nationally representative midwakh survey among young people in UAE and goes beyond simple age- or sex-stratified estimates of midwakh use. Its main weakness is its cross-sectional design and hence our inability to assess changes in prevalence and in sociodemographic correlates over time. Some questions had answer categories that were not insightful, such as many midwakh smoking locations being “other”, which presents opportunities for refinement in future GYTS waves.

### Implications

This study provides evidence that midwakh use is prevalent across UAE students of differing nationalities, including among non-Arabs. Anecdotal review of websites suggests an international midwakh market that is growing [21], and so the spread of midwakh beyond the UAE is something the public health and tobacco control communities should be mindful of. Far more research is warranted into midwakh in the UAE, including continued surveillance, reasons for use, its interaction with other tobacco products and risky behaviours–all by population subgroups. The apparent absence of qualitative literature is a research priority to understand reasons and context of midwakh use. While continued efforts on these and information on the possible adverse health effects of midwakh are gathered, it should not delay policy discourse and implementation. That health warning recall is low and underage sales is common are a cause for action. Further studies should assess the price elasticity of demand midwakh tobacco and its cross-price elasticity with respect to other tobacco products. Full application of the Framework Convention on Tobacco Control to midwakh should be a priority for the UAE.

## Supporting information

S1 Table Midwakh measures. (DOCX)
