# Supplementary material for: Prevalence, attitudes, behaviours and policy evaluation of midwakh smoking among young people in the United Arab Emirates: Cross-sectional analysis of the Global Youth Tobacco Survey
Source: PLoS One. 2019 Apr 24;14(4):e0215899. doi: 10.1371/journal.pone.0215899 (PMC6481845; doi:10.1371/journal.pone.0215899)
Supplement: S1 Table — (DOCX) [file pone.0215899.s001.docx]

**S1 Table. Midwakh measures**

| Measure | Response categories | Definitions for this study |
| --- | --- | --- |
| Prevalence |  |  |
| During the past 30 days, on how many days did you smoke medwakh? | 0 days; 1 or 2 days; 3 to 5 days; 6 to 9 days; 10 to 19 days; 20 to 29 days; All 30 days | Current use: any response other than “0 days”  Daily use: “All 30 days” |
| Have you ever tried or experimented with medwakh, even if one or two puffs? | Yes; No | Ever use: “Yes” |
| Please think about the days you smoked medwakh during the past 30 days. How many medwakh did you usually smoke per day? | I did not smoke medwakh during the past 30 days; Less than 1 medwakh per day; 1 medwakh per day; 2 to 5 medwakh per day; 6 to 10 medwakh per day; 11 to 20 medwakh per day; More than 20 medwakh per day | Number of midwakhs per month: median of each response category “number of days smoked per month” multiplied by the median of each response category of “number smoked per day”; variable considered continuous |
| How old were you when you first tried smoking medwakh? | I have never tried smoking medwakh; 7 years old or younger; 8 or 9 years old; 10 or 11 years old; 12 or 13 years old; 14 or 15 years old; 16 years old or older | Age of first use: median of each response category and variable considered continuous |
|  |  |  |
| Cessation |  |  |
| Do you want to stop smoking medwakh now? | I have never smoked medwakh; I don’t smoke medwakh now; Yes; No | Intention to quit: “Yes”, and analysis restricted to ever users |
| During the past 12 months, did you ever try to stop smoking medwakh? | I have never smoked medwakh; I did not smoke medwakh during the past 12 months; Yes; No | Quit attempt: “Yes”, and analysis restricted to ever users |
|  |  |  |
| Attitudes |  |  |
| Do you think the smoke from other people’s medwakh is harmful to you? | Definitely not; Probably not; Probably yes; Definitely yes | Reduced perception of susceptibility to second-hand smoke: “Definitely not” or “Probably not” |
| If one of your best friends offered you medwakh, would you smoke it? | Definitely not; Probably not; Probably yes; Definitely yes | Increased peer-influenced propensity to smoke: “Definitely yes” or “Probably yes” |
| Once someone has started smoking medwakh, do you think it would be difficult for them to quit? | Definitely not; Probably not; Probably yes; Definitely yes | Increased perceived ease of quitting: “Definitely not” or “Probably not” |
| Do you think smoking medwakh helps people feel more comfortable or less comfortable at celebrations, parties, or in other social gatherings? | More comfortable; less comfortable; No difference whether smoking medwakh or not | Increased perceived comfort: “More comfortable” |
| Do you agree or disagree with the following: “I think I might enjoy smoking medwakh” | I currently smoke medwakh; Strongly agree; Agree; Disagree; Strongly disagree | Increased perceived enjoyment: “Strongly agree”, “Agree”, and analysis restricted to those who answered anything other than “I currently smoke medwakh” |
|  |  |  |
| Accessibility |  |  |
| The last time you smoked medwakh during the past 30 days, how did you get it? | I did not smoke medwakh in the last 30 days; I bought them in a medwakh store or shop (grocery); I bought it from a gas station; I bought it at a supermarket; I bought it from a cafeteria; I got it from someone else; I got it some other way | Attainment method: response categories used verbatim, and analysis restricted to past-30 day) users |
| The last time you smoked medwakh during the past 30 days, where did you smoke it? | I did not smoke medwakh in the last 30 days; at home; at a coffee shop; at a restaurant; at a club; school; other | Smoking location: response categories used verbatim, and analysis restricted to past-30 day) users |
|  |  |  |
| Policy |  |  |
| During the past 30 days, did anyone refuse to sell you medwakh because of your age? | I did not try to buy medwakh during the past 30 days; Yes, someone refused to sell me medwakh because of my age; No, my age did not keep me from buying medwakh | Refusal of purchase due to age: “Yes”, and analysis restricted to past-30 day users |
| During the past 30 days, did you see any health warnings on medwakh tobacco packages? | Yes, but I didn’t think much of them; Yes, and the led me to think about quitting medwakh smoking or not starting medwakh smoking; No | Health warning recall: Any response including “Yes” |
|  |  |  |

Note: GYTS uses the spelling “medwakh” rather than “midwakh”
